# Medication literacy among patients with chronic diseases in long-term care facilities: a latent profile analysis

**DOI:** 10.3389/fpubh.2025.1721259

**Published:** 2026-01-14

**Authors:** Qiongyao Feng, Qiu Yang, Yingchao Guo, Jinfeng Jiang

**Affiliations:** 1 Nursing Department, Affiliated Hospital of North Sichuan Medical College, Nanchong, Sichuan, China; 2 Department of Endocrinology, Affiliated Hospital of North Sichuan Medical College, Nanchong, Sichuan, China

**Keywords:** chronic disease, latent profile analysis, long-term care facility, medication literacy, the older

## Abstract

**Objective:**

This study aims to examine the potential medication literacy profiles of patients with chronic diseases in long-term care facilities and to analyze the influencing factors, thereby providing a basis for developing targeted intervention programs.

**Methods:**

This study conducted a cross-sectional study among 403 older patients with chronic diseases in 41 long-term care facilities in Nanchong City, China, from January to April 2025. Latent profile analysis was conducted using the 23 items of the older adults chronic disease medication literacy scale as manifest variables, followed by multinomial logistic regression to analyze the influencing factors.

**Results:**

Three distinct medication literacy profiles were identified: high medication literacy with active communication and interaction (30.3%), moderate medication literacy with passive dependence (47.4%), and low medication literacy with limited information acquisition (22.3%). Multinomial logistic regression analysis revealed that educational level, pension status, frequency of health checkups, staff attention, self-assessment of medication effectiveness, perceived social support, and self-efficacy for appropriate medication use significantly influenced the medication literacy categories.

**Conclusion:**

The overall medication literacy of patients with chronic diseases in long-term care facilities is above average; however, significant individual differences remain. Clinical staff and institutional caregivers should develop and implement targeted interventions based on influencing factors to enhance medication literacy.

## 1 Introduction

The Healthy China Initiative (2019–2030) identifies chronic non-communicable diseases as the primary causes of death and disease burden among Chinese residents (1). Moreover, the prevalence of chronic diseases among older individuals in long-term care (LTC) facilities in China exceeds the national average (2). LTC facilities in China are broadly classified into two categories: those with integrated medical and long-term care services and those without. The former includes models such as contractual partnerships between separate entities, the addition of medical services to existing care facilities, or the expansion of long-term care services within medical institutions. In the contractual partnership model, medical institutions provide healthcare services through regular visits, whereas the LTC facilities themselves lack on-site medical staff and thus are unable to provide professional medication management. In models whereby care facilities add medical services or medical institutions add long-term care, standardized medication management services for older residents are still lacking. Although capable of providing medical services, these institutions face resource constraints, leading staff to prioritize critically ill patients. Additionally, many LTC facilities implement a tiered management system whereby residents with better self-care abilities pay less and receive fewer services. Consequently, most chronic disease patients capable of self-care are responsible for managing their own medications. In non-integrated LTC facilities, medication management is primarily the responsibility of the residents themselves and care aides without formal medical training. As a result, the medication literacy (ML) of these residents directly determines their medication safety. Therefore, neither integrated nor non-integrated LTC facilities currently adequately address the daily medication management needs of chronic disease patients. Furthermore, older patients with chronic diseases in LTC facilities are typically of advanced age, have prolonged disease durations, and exhibit complex conditions. They are characterized by high medication utilization rates and frequent polypharmacy, which pose substantial medication safety risks (3).

Medication literacy refers to an individual’s ability to obtain, understand, and use medication information safely and appropriately (4). Studies (5, 6) have shown that ML positively predicts patient medication safety. Patients with low ML often struggle to correctly interpret medication information and to follow professional guidance effectively, leading to a higher incidence of adverse medication events and an increased risk of readmission. Accurately assessing ML helps quantify individual medication abilities and enhances medication safety management (7). Perceived social support refers to assistance received from external sources such as family, friends, and significant others (8). A study among hypertensive patients found that greater social support was associated with improved medication adherence (9). Self-efficacy for appropriate medication use refers to a patient’s confidence in their ability to adhere to a medication regimen for better health outcomes (10). Assessing self-efficacy provides insights into patients’ understanding of pharmacotherapy, willingness to manage medications proactively, and capacity to address medication-related challenges. Therefore, we hypothesize that social support and self-efficacy in appropriate medication use influence ML levels.

Latent profile analysis (LPA) is a person-centered analytical method. Compared to traditional variable-centered approaches, LPA identifies homogeneous subgroups within a population by using categorical latent variables to explain the relationships among observed continuous variables (11). As existing ML research has primarily focused on community and hospital populations (12–14), limited attention has been given to older LTC patients with chronic conditions. Therefore, this study employed LPA to classify the ML of patients with chronic diseases in LTC facilities, explore the characteristics and heterogeneity of different groups, identify the factors influencing distinct ML profiles, and provide a basis for designing targeted intervention programs.

## 2 Subjects and methods

### 2.1 Study participants

From January to April 2025, a total of 403 older patients with chronic diseases were recruited from 41 LTC facilities in Nanchong City. Inclusion criteria: (1) aged 60 years or older; (2) residing in LTC facilities for at least 3 months; (3) diagnosed with a chronic non-communicable disease (e.g., hypertension, diabetes, hyperlipidemia, hyperuricemia, gout, asthma, chronic obstructive pulmonary disease, cancer, etc.); and (4) conscious, with no communication or language barriers. Exclusion criteria: (1) severe illness or terminal stage of disease; (2) cognitive impairment, as determined by the Clock Drawing Test, or other mental disorders; and (3) participation in other clinical studies that may interfere with this research. All subjects in this study agreed to participate and signed the informed consent form voluntarily.

### 2.2 Research tools

#### 2.2.1 General information questionnaire

This questionnaire was self-developed and contains 15 items, which included sociodemographic characteristics (gender, age, educational level, marital status, religious beliefs, type of LTC facility, pension status, family financial capacity, frequency of health checkups, staff attention, self-assessment of medication effectiveness, number of surviving children, number of occupants in the bedroom) and clinical variables (number of chronic diseases, duration of continuous medication use).

#### 2.2.2 Older adults chronic disease medication literacy scale

This scale, developed by Zhao (15), assesses four dimensions: information acquisition, medication knowledge, communication and interaction, and critical thinking, consisting of 23 items. It employs a 5-point Likert scale, with scores ranging from “cannot” to “completely can,” assigned values of 1 to 5, respectively. Higher scores reflect better ML in older patients with chronic diseases. The Cronbach’s α coefficient for the scale in this study was 0.946.

#### 2.2.3 Perceived social support scale

This scale was developed by Zimet et al. (8) and later translated into Chinese by Jiang (16). It consists of three dimensions: family support, friend support, and other support, comprising 12 items. The scale employs a 7-point Likert format, with responses ranging from “strongly disagree” to “strongly agree,” and scores from 1 to 7. Higher scores reflect a higher level of perceived social support. The Cronbach’s α coefficient for the scale in this study was 0.882.

#### 2.2.4 Self-efficacy for appropriate medication use scale

The scale, developed by Risser et al. (17), includes two dimensions: confidence in medication use under difficult conditions and confidence in medication use under uncertain conditions, consisting of 13 items. It uses a 3-point Likert scale, with responses ranging from “no confidence” to “very confident,” scored from 1 to 3. Higher scores reflect greater self-efficacy in adhering to appropriate medication use. Chinese scholars Dong et al. (18) adapted the scale for Chinese use and evaluated its validity. In this study, the Cronbach’s α coefficient for the scale was 0.799.

### 2.3 Pilot survey

A pilot survey was conducted from October to November 2024. A convenience sample of 25 chronic disease patients who met the inclusion and exclusion criteria was recruited from three LTC facilities. The purpose was to evaluate the reliability and applicability of the questionnaire, assess the comprehensiveness of its content and the clarity of its wording, and test its reliability and validity. The questionnaire was subsequently revised and refined based on this feedback.

### 2.4 Sample size calculation

The sample size was calculated using the formula for cross-sectional studies (19):

$$N={\left(\frac{U_{\alpha }\times S}{\delta }\right)}^{2}$$

Where the significance level α was set at 0.05 (*U*_α_ = 1.96), and the margin of error δ was 2. A pilot survey of 25 chronic disease patients in LTC facilities showed a standard deviation (S) of 19.3 for the total ML score. Accounting for a potential 5% invalidity rate, the minimum required sample size was 377. Due to a slightly higher number of eligible older residents in the participating institutions than anticipated, along with good survey cooperation, the final sample size obtained slightly exceeded the calculated requirement. This study ultimately included 403 participants.

### 2.5 Data collection

Information about LTC facilities in the city was obtained from the Nanchong Municipal Government website. A total of 246 facilities were included, divided into three groups by size using stratified sampling: small (≤50 beds), medium (51–100 beds), and large (≥101 beds). Ten to fifteen LTC facilities were selected from each group. To obtain institutional approval, the directors of the sampled facilities were contacted via telephone using official contact information. During these calls, the research team elaborated on the study’s purpose, methodology, procedures, and data privacy protection measures. For facilities that provided preliminary consent, a survey date and time were mutually agreed upon. On the day of data collection, institutional informed consent was secured prior to the commencement of any surveys. If a facility declined to participate, it was replaced by a substitute facility selected from the same stratum. A total of 41 LTC facilities were surveyed, and 413 questionnaires were collected. Ten invalid questionnaires with logical contradictions or incorrect content were discarded, leaving 403 valid questionnaires, with a valid recovery rate of 97.6%.

### 2.6 Data analysis

Data analysis was conducted using Mplus 8.3 and SPSS 27.0 software. LPA of ML among patients with chronic diseases in LTC facilities was performed using Mplus 8.3. The analysis started with one category and progressively added more categories, comparing model fit indices to evaluate differences and select the optimal model. A better model fit is indicated by smaller values of the Akaike information criterion (AIC), Bayesian information criterion (BIC), and adjusted Bayesian information criterion (aBIC) (20, 21). Information entropy, ranging from 0 to 1, measures classification accuracy, with values closer to 1 indicating higher accuracy (22). When Entropy > 0.8, it suggests that the model’s classification accuracy exceeds 90% (23). The Lo–Mendell–Rubin adjusted likelihood ratio test (LMRT) and Bootstrapped likelihood ratio test (BLRT) were used to compare the fit differences between potential category models (24). A *p*-value < 0.05 suggests that the k-category model is superior to the (k-1)-category model (25). Data analysis was conducted using SPSS 27.0. Quantitative data that did not follow a normal distribution were expressed as the median (interquartile range) [M (IQR)], with intergroup comparisons conducted using the Kruskal-Wallis *H* test. Qualitative data were expressed as frequencies, percentages, or rates (%), with intergroup comparisons performed using the chi-square test and Fisher’s exact test. Multinomial logistic regression was applied to explore factors influencing different latent profiles of ML. A *p*-value < 0.05 was considered statistically significant.

## 3 Results

### 3.1 General information on study subjects

General information about 403 patients with chronic diseases in LTC facilities is presented in Table 1.

**Table 1:** Univariate analysis of participants’ demographics and the three latent medication literacy profiles.

Variables	Categories	Low ML	Moderate ML	High ML	Statistics	*p*
		*n* = 90	*n* = 191	*n* = 122		
		22.3%	47.4%	30.3%		
		*n* (%) OR M (IQR)	*n* (%) OR M (IQR)	*n* (%) OR M (IQR)		
Gender	Male	32 (35.6%)	64 (33.5%)	47 (38.5%)	*χ*^2^ *=* 0.819	0.664
	Female	58 (64.4%)	127 (66.5%)	75 (61.5%)		
Age group	<80 years	30 (33.3%)	36 (18.8%)	23 (18.9%)	*χ*^2^ *=* 8.522	0.014
	≥80 years	60 (66.7%)	155 (81.2%)	99 (81.1%)		
Education level	Elementary school and below	73 (81.1%)	148 (77.5%)	47 (38.5%)	*χ*^2^ *=* 61.835	<0.001
	Junior high school and above	17 (18.9%)	43 (22.5%)	75 (61.5%)		
Marital status	Married	6 (6.7%)	18 (9.4%)	35 (28.7%)	*χ*^2^ *=* 28.004	<0.001
	Unmarried	84 (93.3%)	173 (90.6%)	87 (71.3%)		
Religious beliefs	Yes	6 (6.7%)	19 (9.9%)	14 (11.5%)	*χ*^2^ *=* 1.4	0.496
	None	84 (93.3%)	172 (90.1%)	108 (88.5%)		
Type of LTC facility	Integrated medical and long-term care	29 (32.2%)	89 (46.6%)	72 (59.0%)	*χ*^2^ *=* 14.966	<0.001
	Non-integrated	61 (67.8%)	102 (53.4%)	50 (41.0%)		
Duration of continuous medication use	≤5 years	57 (63.3%)	62 (32.5%)	30 (24.6%)	*χ*^2^ *=* 36.667	<0.001
	5–10 years	10 (11.1%)	43 (22.5%)	29 (23.8%)		
	>10 years	23 (25.6%)	86 (45.0%)	63 (51.6%)		
Pension status	Yes	25 (27.8%)	68 (35.6%)	95 (77.9%)	*χ*^2^ *=* 70.022	<0.001
	None	65 (72.2%)	123 (64.4%)	27 (22.1%)		
Family financial capacity	No pressure	38 (42.2%)	96 (50.3%)	92 (75.4%)	*χ*^2^ *=* 32.975	<0.001
	Under some pressure	42 (46.7%)	87 (45.5%)	27 (22.1%)		
	Unaffordable	10 (11.1%)	8 (4.2%)	3 (2.5%)		
Frequency of health checkups	Regular	3 (3.3%)	23 (12.0%)	51 (41.8%)	*χ*^2^ *=* 79.773	<0.001
	Occasional	60 (66.7%)	139 (72.8%)	68 (55.7%)		
	Never	27 (30.0%)	29 (15.2%)	3 (2.5%)		
Staff attention	Very concerned	46 (51.1%)	142 (74.3%)	94 (77.0%)	*χ*^2^ *=* 24.960	<0.001
	Partial concerned	19 (21.1%)	31 (16.2%)	16 (13.1%)		
	Rarely concerned	25 (27.8%)	18 (9.4%)	12 (9.8%)		
Self-assessment of medication effectiveness	Good	45 (50%)	132 (69.1%)	108 (88.5%)	/	<0.001*
	Neutral	42 (46.7%)	56 (29.3%)	10 (8.2%)		
	Bad	3 (3.3%)	3 (1.6%)	4 (3.3%)		
Number of surviving children		2 (1)	2 (1)	2 (1)	*H* = 1.315	0.518
Number of occupants in the bedroom		2 (0)	2 (0)	2 (0)	*H* = 4.349	0.114
Number of chronic diseases		1 (1)	2 (2)	2 (1)	*H* = 18.819	<0.001
PSSS		4.25 (1)	5.25 (1)	5.92 (1)	*H* = 151.385	<0.001
SEAMS		2.27 (1)	2.54 (0)	2.77 (0)	*H* = 71.806	<0.001

ML, Medication Literacy; PSSS, Perceived Social Support Scale; SEAMS, Self-efficacy for Appropriate Medication Use Scale.

^*^Fisher’s exact test.

### 3.2 Potential profile classification and model fitting

**Figure 1:**
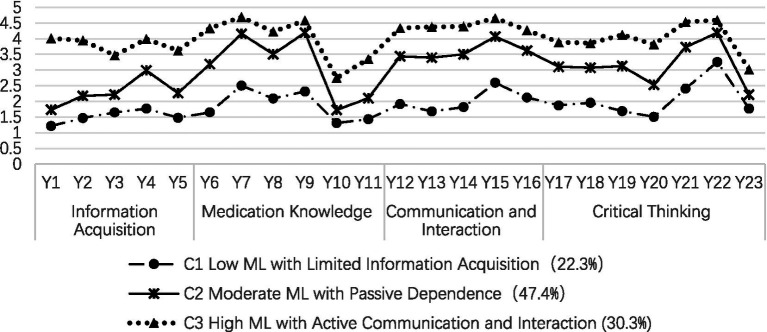
Three latent profile distributions of medication literacy among chronic disease patients in long-term care facilities.

The 23 items of the older adults chronic disease medication literacy scale (OACD-MLS) were used as manifest variables for LPA, with 1–4 latent profile models being fitted, as shown in Table 2. As the number of model categories increased, the AIC, BIC, and aBIC values decreased progressively, reaching a minimum at the 4-category model. However, the LMRT test yielded no statistically significant results. For the 3-category model, the Entropy value was 0.948, exceeding 0.8, indicating classification accuracy greater than 90%. The *p*-values for the LMR and BLRT tests were both <0.05, suggesting that the 3-category model was superior to the 2-category model. The average classification rates for the three categories were 97.5, 97.9, and 97.5%, respectively, confirming the reliability of the 3-category model fit. Consequently, the 3-category model was chosen as the optimal model for the current sample. The characteristics of the latent profiles of ML in chronic disease patients in LTC facilities are shown in Figure 1. The categories were named based on the manifest characteristics of each scale item and their practical significance. Among these, C1 scores were generally low, particularly for items related to information acquisition ability, significantly differing from the other two groups. This category was named “low medication literacy with limited information acquisition type” and represented 22.6% of the total sample. C3 scores were generally high, with the highest scores in items related to communication and interaction ability, and were named “high medication literacy with active communication and interaction type,” representing 30.3% of the total sample. C2 falls between C3 and C1. Patients in this category possess some drug knowledge but score significantly lower than the C3 group on critical thinking ability items, suggesting a tendency to rely on external guidance. Therefore, it was named “moderate medication literacy with passive dependency type,” representing 47.1% of the total sample.

**Table 2:** Potential profile model fitting indicators for medication literacy.

Mode	AIC	BIC	a BIC	Entropy	*P* LMRT	*P* BLRT	Category probability
1	30539.414	30723.365	30577.402	/	/	/	/
2	27567.996	27847.922	27625.805	0.961	<0.001	<0.001	0.32/0.68
**3**	**26378.695**	**26754.595**	**26456.323**	**0.948**	**<0.001**	**<0.001**	**0.23/0.47/0.30**
4	25975.765	26447.639	26073.213	0.931	0.171	<0.001	0.22/0.39/0.12/0.27

Bold values indicate the optimal profile model selected in this study.

### 3.3 Factors influencing medication literacy

The results of the univariate analysis showed that the distribution of the three potential categories of ML differed significantly (*p* < 0.05) in the following variables: age, education level, marital status, type of LTC facility, duration of continuous medication use, pension status, family financial capacity, frequency of health checkups, staff attention, self-assessment of medication effectiveness, number of chronic diseases, perceived social support score, and self-efficacy in rational medication use, as shown in Table 1. These significant variables were included as independent variables in a multinomial logistic regression model, with their coding schemes detailed in Table 3. The results of this analysis, which modeled the three potential categories as the dependent variable, are presented in Table 4.

**Table 3:** Independent variable assignment method.

Independent variable	Assignment method
Age group	1 represents <80 years; 2 represents ≥80 years
Education level	1 represents junior high school and above; 2 represents elementary school and below
Marital status	1 represents married; 2 represents unmarried
Type of LTC facility	1 represents integrated medical and long-term care; 2 represents non- integrated
Duration of continuous medication use	1 represents ≤5 years; 2 represents 5–10 years; 3 represents >10 years
Pension status	1 represents having a pension; 2 represents no pension
Family financial capacity	1 represents no pressure; 2 represents under some pressure; 3 represents unaffordable
Frequency of health checkups	1 represents regular health checkups; 2 represents occasional health checkups; 3 represents never health checkups
Staff attention	1 represents very concerned 2 represents partial Very concerned; 3represents rarely concerned
Self-assessment of medication effectiveness	1 represents good; 2 represents neutral; 3 represents bad
Number of chronic diseases	Original value input
Perceived social support scale	Original value input
Self-efficacy for appropriate medication use scale	Original value input

**Table 4:** Results of a multinomial logistic regression analysis of potential factors influencing medication literacy among chronic disease patients in long-term care facilities.

Comparison	Predictors	β	*p*	OR	95%*CI*
C^a^	Constant	−19.371	<0.001		
	Educational level: junior high school and above	1.181	0.021	3.259	1.193 ~ 8.899
	Have a pension	1.702	<0.001	5.487	2.031 ~ 14.827
	Regular health checkup	2.508	0.019	12.283	1.523 ~ 99.075
	PSSS	2.099	<0.001	8.157	4.217 ~ 15.779
	SEAMS	2.53	<0.001	12.552	3.236 ~ 48.690
C^b^	Constant	−9.829	<0.001		
	Educational level: junior high school and above	1.000	0.003	2.719	1.409 ~ 5.247
	Have a pension	1.297	<0.001	3.66	1.767 ~ 7.579
	Regular health checkup	1.891	0.015	6.627	1.441 ~ 30.481
	Self-assessment of medication effectiveness: neutral	−2.208	0.032	0.11	0.015 ~ 0.827
	PSSS	0.845	0.001	2.328	1.404 ~ 3.860
	SEAMS	1.114	0.046	3.045	1.018 ~ 9.109
B^a^	Constant	−9.542	<0.001		
	Staff attention to patients: very concerned	0.875	0.049	2.399	1.005 ~ 5.732
	PSSS	1.254	<0.001	3.504	2.173 ~ 5.648
	SEAMS	1.416	0.003	4.122	1.595 ~ 10.625

C^a^ High medication literacy with active communication and interaction versus low medication literacy with limited information acquisition. Reference: low medication literacy with limited information acquisition.

C^b^ High medication literacy with active communication and interaction versus moderate medication literacy with passive dependence. Reference: moderate medication literacy with passive dependence.

B^a^ Moderate medication literacy with passive dependence versus low medication literacy with limited information acquisition. Reference: low medication literacy with limited information acquisition.

The three latent categories were treated as dependent variables, with statistically significant variables from the univariate analysis included in the multinomial logistic regression model. The independent variables were coded as shown in Table 3. The results of the multinomial logistic regression analysis are presented in Table 4.

## 4 Discussion

### 4.1 Patients with chronic diseases in long-term care facilities exhibit above-average medication literacy, but inadequate risk awareness and coping skills

This study found that 30.3% of patients in LTC facilities exhibited high ML with active communication and interaction, 47.1% had moderate ML with passive dependence, and 22.6% had low ML with limited information acquisition. These findings suggest that ML in this population is moderately high, surpassing that of the general population, as reported by Ni et al. (26). This discrepancy may be due to differences in the study population and research tools. In this study, 47% of participants were from LTC facilities that integrate medical care with long-term services, which typically offer professional medication support, such as health lectures, doctor visits, and monitoring, thereby enhancing medication knowledge. Even in facilities without integrated care, patients receive services such as medication reminders and basic assistance, which help ensure medication adherence and safety to some extent. Additionally, different measurement tools may also influence the classification outcomes. It is worth noting that all three patient groups scored poorly on items 10 (Do you know the adverse reactions of the drugs you are currently taking?), 11 (Do you know the expiration date of the drugs you are currently taking?), and 23 (Do you know the correct way to handle medication errors or missed doses?), which indicates safety concerns in their medication risk awareness and response abilities. Possible reasons include that healthcare providers often focus on routine instructions, such as medication timing and dosage, while neglecting to educate patients on risk awareness and response strategies. Additionally, cognitive inertia resulting from prolonged medication use in some older individuals reduces their proactive risk awareness. This emphasizes the importance of comprehensive medication safety education. During patient education, both proper medication use and the development of risk identification and response skills should be emphasized to help older patients fully understand medication safety. Integrated medical and long-term care institutions should further transform their service philosophy, shifting from medication safety supervision to empowerment-based risk management. This can be achieved by establishing a standardized process that includes risk assessment, identification, and response, thereby integrating risk education into all aspects of daily care. Non-integrated institutions, on the other hand, need to focus on strengthening training for care aides. Through methods such as case reviews and scenario simulations, they should enhance the aides’ ability to guide patients in identifying and responding to common medication risks, thereby comprehensively improving the patients’ risk identification and response skills.

### 4.2 Medication literacy varies significantly among chronic disease patients in long-term care facilities

LPA revealed significant individual differences in profiles among chronic disease patients in LTC facilities, who were categorized into three distinct types: (1) Low ML with Limited Information Acquisition: 90 patients (22.6%) scored 43.23 ± 9.64 on the OACD-MLS. Patients in this category scored poorly across all items, especially in information acquisition, suggesting difficulty in obtaining reliable medication information. Previous studies (27, 28) have shown that clear, conspicuous labels, large text, and bright colors on drug packaging can improve older patients’ understanding of medication use. This suggests that modifying drug packaging in age-appropriate ways can effectively convey key medication knowledge. Meanwhile, it is recommended that all LTC facilities strengthen medication assistance functions to help overcome these information comprehension barriers. Specifically, integrated institutions should leverage their professional teams, with pharmacists or physicians providing clear, visual medication guides. Non-integrated institutions should enhance care staff’s communication skills and introduce intelligent medication management devices. A study by Peng et al. (29) confirmed that intelligent medication management systems can significantly improve medication self-management in older patients, offering a practical solution to compensate for shortages in professional staff; (2) Moderate ML with Passive Dependence: 191 patients (47.1%) scored 70.16 ± 7.29 on the OACD-MLS. Patients in this category possess basic medication knowledge and the ability to discern information but lack self-management initiative and are overly reliant on medical staff and caregivers for medication safety. This suggests the need to improve patients’ awareness that “individuals are primarily responsible for their own health” and encourage their proactive engagement in health management; (3) High ML with Active Communication and Interaction: 122 patients (30.3%) scored 92.93 ± 8.07 on the OACD-MLS. Patients in this category exhibit high ML and strong communication skills, reflecting their ability to actively communicate, accurately understand, and effectively disseminate medication information. LTC facilities are encouraged to develop these patients as key figures in peer education on medication safety, enhancing their risk awareness while preserving their strengths to improve the overall ML of the older adult population.

### 4.3 Patients with junior high school education or higher, receiving a pension, and undergoing regular health checkups are more likely to exhibit high medication literacy with active communication and interaction

This study found that compared to patients with an elementary school education or below, those with at least a junior high school education were more likely to be classified into the high ML with active communication and interaction type (OR = 3.259; OR = 2.719), consistent with previous research findings (30). This may be because patients with higher education have broader knowledge, better access to information, and enhanced ability to understand and process it. Compared to patients without pensions, those with pensions are more likely to belong to the high ML with active communication and interaction type (OR = 5.487; OR = 3.66). Previous studies (31) have shown that ML correlates positively with income. Pensions, as a stable income source, provide financial security for chronic disease patients in LTC facilities, increasing their likelihood of adopting advanced treatments and purchasing necessary medications. In contrast, patients without pensions may reduce or discontinue medication due to financial instability and the burden of LTC, which may lower their ML. Compared to patients who never undergo regular check-ups, those who do are more likely to be classified as having high ML with active communication and interaction type (OR = 12.283; OR = 6.627), similar to the findings of Xu et al. (32). One reason may be that patients who do not have regular check-ups interact less with medical staff, limiting their ability to accurately understand medication-related information. Additionally, prolonged absence from professional medical supervision may reduce patients’ awareness of the importance of drug treatment, thereby increasing the risk of inadequate ML. This underscores the need to focus on patients in LTC facilities with low education, no pensions, and infrequent check-ups. Studies (33) indicate that pictograms can enhance medication management for less educated populations. Therefore, it is recommended that healthcare providers employ pictograms (34) during medication prescribing and discharge counseling, while LTC staff incorporate them into daily medication reminders to enhance patients’ medication knowledge. For instance, key usage instructions, dosages, and precautions for common drugs can be converted into intuitive graphical labels affixed to medication packages, thereby addressing the health information comprehension barriers faced by patients with limited literacy. As LTC insurance has been piloted in several Chinese cities, it is recommended that the government expand coverage, drawing from current pilot experiences (35), to help eligible chronic disease patients in LTC facilities manage economic challenges. In addition, LTC facilities should actively establish coordinated and continuous healthcare service systems that integrate internal and external resources. Integrated institutions should develop two-way referral mechanisms based on health data sharing to ensure patients receive timely specialized care from hospitals when needed. Non-integrated institutions should strengthen collaboration with primary care providers by introducing regular visits from community doctors, conducting health education sessions, and performing medication follow-ups, thereby building sustainable professional support networks for their residents.

### 4.4 Patients who self-assessment of medication effectiveness as neutral and receive high staff attention are more likely to exhibit moderate medication literacy with passive dependency

The results showed that patients who perceived their medication treatment effectiveness as neutral were less likely to be classified as having high ML with active communication and interaction (OR = 0.11) compared to those with moderate ML with passive dependence. This finding aligns with Gao’s study (36). This may be because these patients’ self-rated of medication effectiveness is intermediate, neither reaching the positive evaluation of “good” nor the negative judgment of “poor.” As a result, they show compliance but lack proactivity in medication management, keeping their ML at an intermediate level. It is recommended to promote patient participation in medication decision-making processes in clinical practice to build medication confidence. Simultaneously, LTC facilities of all types should implement appropriate care plans by conducting personalized capacity assessments to stratify the management of older patients. This approach helps avoid creating substitute care for those with better self-care abilities, thereby facilitating their transition from passive dependence to active engagement and ultimately enhancing their ML level. Compared to older individuals with low ML with limited information acquisition, those who felt that LTC staff were highly concerned about their health were more likely to belong to the moderate ML with passive dependence group (OR = 2.399). However, no significant association was observed for the high ML with active communication and interaction group. One explanation is that high levels of staff attention, as a positive emotional experience, encourage older individuals to focus more on their health and medication, improving their ML; however, medication supervision in LTC facilities currently focuses mainly on safety control, lacking a systematic approach to medication education. While this safety control ensures medication safety, it does not effectively improve older patients’ deeper medication knowledge and self-management abilities, ultimately limiting their ability to achieve higher levels of ML. This highlights the substantial potential of LTC facilities in fostering health-promoting environments and recommends reinforcing this supportive effect. Specifically, integrated medical and long-term care institutions should fully utilize multidisciplinary geriatric teams to gain deeper insights into the characteristics of their older residents. By clarifying the respective responsibilities of physicians, nurses, pharmacists, and caregivers in medication management, these institutions can progressively enhance education on rational medication use. Non-integrated institutions could actively leverage the advancements in China’s long-term care nursing education programs (37) by recruiting relevant personnel.

### 4.5 Patients with higher perceived social support and self-efficacy for appropriate medication use generally exhibit higher medication literacy levels

The study found that patients with higher perceived social support had higher ML levels (OR = 8.157; OR = 2.328; OR = 3.504). These findings align with those of other studies on ML (38). This may be because patients with more social support have access to greater material, emotional, and medication-related informational support (39). Therefore, all types of LTC facilities should better understand older patients’ social support needs, help them build and sustain positive social networks, integrate resources like family companionship, friend encouragement, and caregiver support, and offer personalized strategies to enhance ML (40). The study also found that higher self-efficacy correlated with higher ML levels (OR = 12.552; OR = 3.045; OR = 4.122), consistent with the findings of Luan’s study on community-dwelling older patients with chronic comorbidities (41). Patients with low self-efficacy tend to feel overwhelmed by complex medication information and procedures, which hinders their ability to acquire, understand, and apply medication knowledge, thereby lowering their ML. Therefore, boosting patients’ self-efficacy to encourage active participation in medication self-management is essential. It is recommended that non-integrated institutions actively develop peer support programs, organizing regular meetings for patients with similar health conditions to share medication experiences. Through success story sharing and group encouragement, these initiatives can enhance patients’ medication confidence. Integrated institutions could introduce structured scenario-based training. Williams’ study (42) confirmed that interactive interventions based on real-life simulations can effectively improve patients’ confidence in medication management. It is suggested that healthcare professionals guide patients through various medication-related scenarios in a controlled environment, helping them gradually build self-efficacy through successful practical experiences. Furthermore, evidence suggests a significant positive correlation between patients’ self-efficacy and their illness perception (43). Consequently, efforts to improve self-efficacy should concurrently address patients’ subjective perceptions of disease severity and controllability. It is recommended that interventions targeting self-efficacy incorporate an assessment of illness perception, with particular emphasis on guiding patients to modify negative cognitions. This approach helps patients develop a more positive illness perception, thereby establishing a stronger psychological foundation for sustained enhancement of self-efficacy.

## 5 Summary

Three distinct medication literacy profiles were identified among chronic disease patients in long-term care facilities: high medication literacy with active communication and interaction, moderate medication literacy with passive dependence, and low medication literacy with limited information acquisition. These profiles are influenced by factors including educational level, pension status, frequency of health checkups, staff attention, self-assessment of medication effectiveness, perceived social support, and self-efficacy for appropriate medication use. It is recommended that individualized interventions be tailored to patients based on these influencing factors.

## 6 Limitations

This study has the following limitations. First, as a cross-sectional study with samples drawn exclusively from LTC facilities in Nanchong City, the findings should be generalized with caution to other regions. Future research should consider expanding the sample size, conducting multi-center studies, and incorporating longitudinal designs to better understand the dynamic trajectories of ML among chronic disease patients in LTC facilities. Second, to mitigate reporting bias among older patients regarding prescription drugs, health supplements, and self-purchased medications, this study used “number of chronic diseases” and “duration of medication use” as core variables rather than detailing specific types and quantities of medications. This approach may have constrained a deeper exploration of the complex relationship between polypharmacy and ML. It is worth noting that, as part of a larger research program, the mechanisms by which different types of medications influence ML will be further investigated in a subsequent qualitative study phase. Finally, this study did not systematically collect information on patients’ subjective perceptions of their own chronic disease severity, and this factor may also influence medication literacy. Future research could incorporate it into the scope of investigation.

## Data Availability

The datasets presented in this article are not readily available because the data described in this article are subject to access restrictions due to privacy and ethical considerations. The information was collected from older patients suffering from chronic conditions and includes sensitive personal health details, which are protected under confidentiality agreements. In accordance with data protection laws and institutional ethical guidelines, the datasets cannot be shared publicly. However, upon submission of a formal request approved by the relevant ethics committee, this information may be obtained through the corresponding author. Requests to access the datasets should be directed to Qiu Yang, yang0hao123@163.com.
