# Supplementary material for: Medication literacy among patients with chronic diseases in long-term care facilities: a latent profile analysis
Source: Front Public Health. 2026 Jan 14;13:1721259. doi: 10.3389/fpubh.2025.1721259 (PMC12846932; doi:10.3389/fpubh.2025.1721259)
Supplement: Supplementary file 1 [file Table_1.docx]

**Supplementary materials**

**Table 1** Univariate analysis of participants' demographics and the three latent medication literacy profiles.

| **Variables** | **Categories** | **Low ML** | **Moderate ML** | **High ML** | **Statistics** | ***p*** |  |
| --- | --- | --- | --- | --- | --- | --- | --- |
|  |  | **n=90** | **n=191** | **n=122** |  |  |  |
|  |  | **22.3%** | **47.4%** | **30.3%** |  |  |  |
|  |  | **n(%) OR**  **M(IQR)** | **n(%) OR**  **M(IQR)** | **n(%) OR**  **M(IQR)** |  |  |  |
| **Gender** | Male | 32(35.6%) | 64(33.5%) | 47(38.5%) | *χ*^2^***=***0.819 | 0.664 |  |
|  | Female | 58(64.4%) | 127(66.5%) | 75(61.5%) |  |  |  |
| **Age group** | ＜80 years | 30(33.3%) | 36(18.8%) | 23(18.9%) | *χ*^2^***=***8.522 | 0.014 |  |
|  | ≥80 years | 60(66.7%) | 155(81.2%) | 99(81.1%) |  |  |  |
| **Education level** | Elementary school and below | 73(81.1%) | 148(77.5%) | 47(38.5%) | *χ*^2^***=***61.835 | ＜0.001 |  |
|  | Junior high school and above | 17(18.9%) | 43(22.5%) | 75(61.5%) |  |  |  |
| **Marital status** | Married | 6(6.7%) | 18(9.4%) | 35(28.7%) | *χ*^2^***=***28.004 | ＜0.001 |  |
|  | Unmarried | 84(93.3%) | 173(90.6%) | 87(71.3%) |  |  |  |
| **Religious beliefs** | Yes | 6(6.7%) | 19(9.9%) | 14(11.5%) | *χ*^2^***=***1.4 | 0.496 |  |
|  | None | 84(93.3%) | 172(90.1%) | 108(88.5%) |  |  |  |
| **Type of LTC facility** | Integrated medical and long-term care | 29(32.2%) | 89(46.6%) | 72(59.0%) | *χ*^2^***=***14.966 | ＜0.001 | |
|  | Non-integrated | 61(67.8%) | 102(53.4%) | 50(41.0%) |  |  |  |
| **Duration of continuous medication use** | ≤5 years | 57(63.3%) | 62(32.5%) | 30(24.6%) | *χ*^2^***=***36.667 | ＜0.001 | |
|  | 5-10 years | 10(11.1%) | 43(22.5%) | 29(23.8%) |  |  |  |
|  | ＞10 years | 23(25.6%) | 86(45.0%) | 63(51.6%) |  |  |  |
| **Pension status** | Yes | 25(27.8%) | 68(35.6%) | 95(77.9%) | *χ*^2^***=***70.022 | ＜0.001 | |
|  | None | 65(72.2%) | 123(64.4%) | 27(22.1%) |  |  |  |
| **Family financial capacity** | No pressure | 38(42.2%) | 96(50.3%) | 92(75.4%) | *χ*^2^***=***32.975 | ＜0.001 | |
|  | Under some pressure | 42(46.7%) | 87(45.5%) | 27(22.1%) |  |  |  |
|  | Unaffordable | 10(11.1%) | 8(4.2%) | 3(2.5%) |  |  |  |
| **Frequency of health checkups** | Regular | 3(3.3%) | 23(12.0%) | 51(41.8%) | *χ*^2^***=***79.773 | ＜0.001 | |
|  | Occasional | 60(66.7%) | 139(72.8%) | 68(55.7%) |  |  |  |
|  | Never | 27(30.0%) | 29(15.2%) | 3(2.5%) |  |  |  |
| **Staff attention** | Very concerned | 46(51.1%) | 142(74.3%) | 94(77.0%) | *χ*^2^***=***24.960 | ＜0.001 | |
|  | Partial concerned | 19(21.1%) | 31(16.2%) | 16(13.1%) |  |  |  |
|  | Rarely concerned | 25(27.8%) | 18(9.4%) | 12(9.8%) |  |  |  |

**Table 1** (continued )

| **Self-assessment of medication effectiveness** | Good | 45(50%) | 132(69.1%) | 108(88.5%) | / | ＜0.001* | |
| --- | --- | --- | --- | --- | --- | --- | --- |
|  | Neutral | 42(46.7%) | 56(29.3%) | 10(8.2%) |  |  |  |
|  | Bad | 3(3.3%) | 3(1.6%) | 4(3.3%) |  |  |  |
| **Number of surviving children** | | 2（1） | 2（1） | 2（1） | *H*=1.315 | | 0.518 |
| **Number of occupants in the bedroom** | | 2（0） | 2（0） | 2（0） | *H*=4.349 | | 0.114 |
| **Number of chronic diseases** | | 1（1） | 2（2） | 2（1） | *H*=18.819 | | ＜0.001 |
| **PSSS** | | 4.25（1） | 5.25（1） | 5.92（1） | *H*=151.385 | | ＜0.001 |
| **SEAMS** | | 2.27（1） | 2.54（0） | 2.77（0） | *H*=71.806 | | ＜0.001 |

Note. ML, Medication Literacy; PSSS, Perceived Social Support Scale; SEAMS, Self-efficacy for Appropriate Medication Use Scale.

* Fisher’s exact test

| **Table 2** Potential profile model fitting indicators for medication literacy. | | | | | | | |
| --- | --- | --- | --- | --- | --- | --- | --- |
| Mode | AIC | BIC | a BIC | Entropy | *P* LMRT | *P* BLRT | Category probability |
| 1 | 30539.414 | 30723.365 | 30577.402 | / | / | / | / |
| 2 | 27567.996 | 27847.922 | 27625.805 | 0.961 | ＜0.001 | ＜0.001 | 0.32/0.68 |
| **3** | **26378.695** | **26754.595** | **26456.323** | **0.948** | **＜0.001** | **＜0.001** | **0.23/0.47/0.30** |
| 4 | 25975.765 | 26447.639 | 26073.213 | 0.931 | 0.171 | ＜0.001 | 0.22/0.39/0.12/0.27 |

Note. Bold values indicate the optimal profile model selected in this study.

**Table 3** Independent variable assignment method.

| **Independent Variable** | **Assignment Method** |
| --- | --- |
| **Age group** | 1 represents ＜80 years ; 2 represents ≥80 years |
| **Education level** | 1 represents junior high school and above; 2 represents elementary school and below |
| **Marital status** | 1 represents married; 2 represents unmarried |
| **Type of LTC facility** | 1 represents integrated medical and long-term care; 2 represents non- integrated |
| **Duration of continuous medication use** | 1 represents ≤5 years; 2 represents 5-10 years; 3 represents >10 years |
| **Pension status** | 1 represents having a pension; 2 represents no pension |
| **Family financial capacity** | 1 represents no pressure; 2 represents under some pressure; 3 represents unaffordable |
| **Frequency of health checkups** | 1 represents regular health checkups; 2 represents occasional health checkups; 3 represents never health checkups |
| **Staff attention** | 1 represents very concerned 2 represents partial Very concerned; 3represents rarely concerned |
| **Self-****assessment of medication effectiveness** | 1 represents good; 2 represents neutral; 3 represents bad |
| **Number of chronic diseases** | Original value input |
| **Perceived Social Support Scale** | Original value input |
| **Self-efficacy for Appropriate Medication Use Scale** | Original value input |

**Table 4** Results of a multinomial logistic regression analysis of potential factors influencing medication literacy among chronic disease patients in long-term care facilities.

| **Comparison** | **Predictors** | ***β*** | ***p*** | **OR** | **95%*CI*** |
| --- | --- | --- | --- | --- | --- |
| C^a^ | **Constant** | -19.371 | ＜0.001 |  |  |
|  | **Educational level: junior high school and above** | 1.181 | 0.021 | 3.259 | 1.193~8.899 |
|  | **Have a pension** | 1.702 | ＜0.001 | 5.487 | 2.031~14.827 |
|  | **Regular health checkup** | 2.508 | 0.019 | 12.283 | 1.523~99.075 |
|  | **PSSS** | 2.099 | ＜0.001 | 8.157 | 4.217~15.779 |
|  | **SEAMS** | 2.53 | ＜0.001 | 12.552 | 3.236~48.690 |
| C^b^ | **Constant** | -9.829 | <0.001 |  |  |
|  | **Educational level: junior high school and above** | 1.000 | 0.003 | 2.719 | 1.409~5.247 |
|  | **Have a pension** | 1.297 | <0.001 | 3.66 | 1.767~7.579 |
|  | **Regular health checkup** | 1.891 | 0.015 | 6.627 | 1.441~30.481 |
|  | **Self-assessment of medication effectiveness:** **neutral** | -2.208 | 0.032 | 0.11 | 0.015~0.827 |
|  | **PSSS** | 0.845 | 0.001 | 2.328 | 1.404~3.860 |
|  | **SEAMS** | 1.114 | 0.046 | 3.045 | 1.018~9.109 |
| B^a^ | **constant** | -9.542 | ＜0.001 |  |  |
|  | **Staff attention to patients: very concerned** | 0.875 | 0.049 | 2.399 | 1.005~5.732 |
|  | **PSSS** | 1.254 | <0.001 | 3.504 | 2.173~5.648 |
|  | **SEAMS** | 1.416 | 0.003 | 4.122 | 1.595~10.625 |

Note. C^a^ High medication literacy with active communication and interaction versus low medication literacy with limited information acquisition. Reference: low medication literacy with limited information acquisition.

C^b^ High medication literacy with active communication and interaction versus moderate medication literacy with passive dependence. Reference: moderate medication literacy with passive dependence.

B^a^ Moderate medication literacy with passive dependence versus low medication literacy with limited information acquisition. Reference: low medication literacy with limited information acquisition.


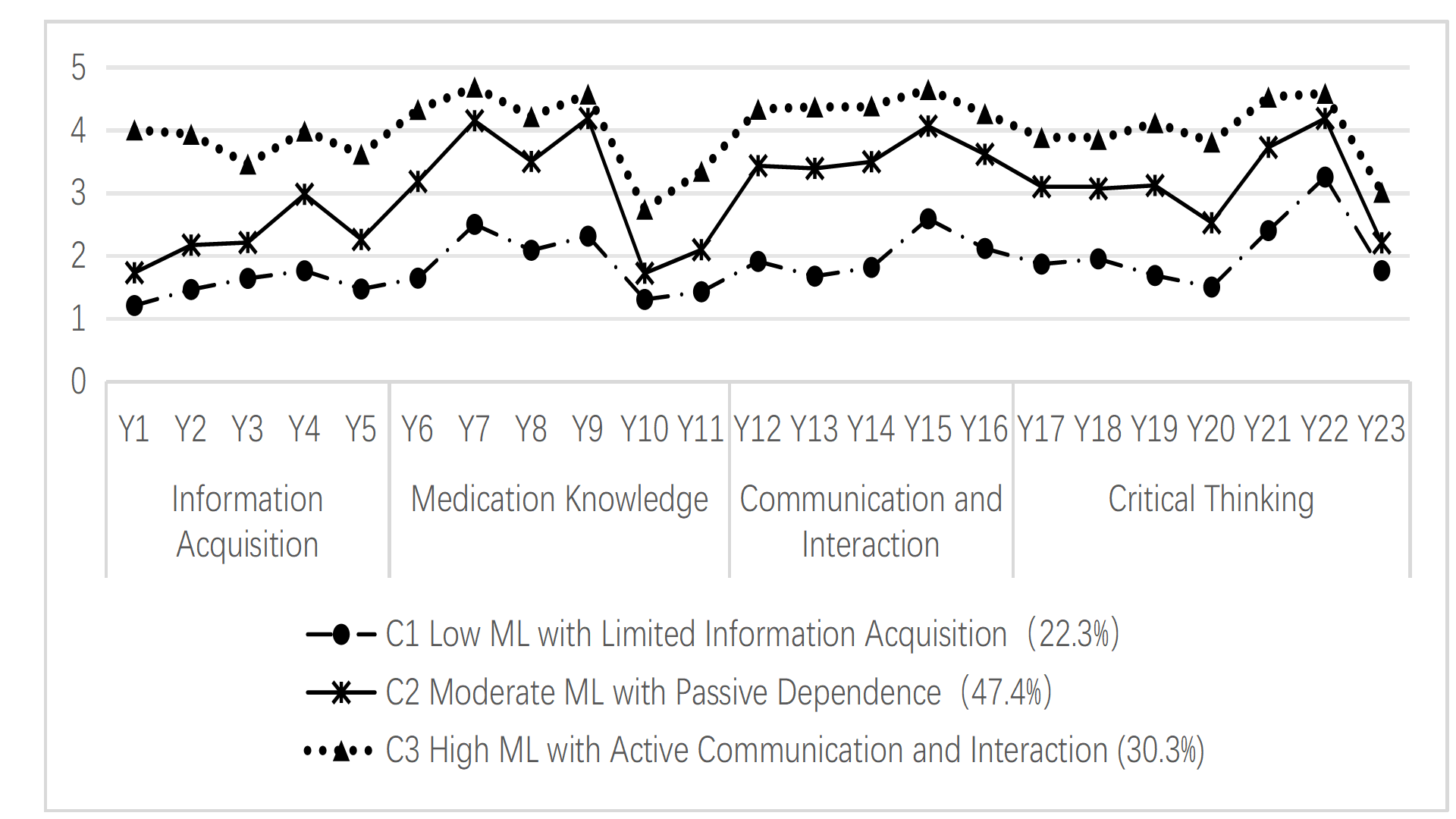


**Fig.1.** Three latent profile distributions of medication literacy among chronic disease patients in long-term care facilities.
